# Eriodictyol and Homoeriodictyol Improve Memory Impairment in Aβ_25–35_-Induced Mice by Inhibiting the NLRP3 Inflammasome

**DOI:** 10.3390/molecules27082488

**Published:** 2022-04-12

**Authors:** Pengli Guo, Mengnan Zeng, Shengchao Wang, Bing Cao, Meng Liu, Yuhan Zhang, Jufang Jia, Qinqin Zhang, Beibei Zhang, Ru Wang, Xiaoke Zheng, Weisheng Feng

**Affiliations:** 1 College of Pharmacy, Henan University of Chinese Medicine, 156 Jinshui East Road, Zhengzhou 450046, China; guopl1998@163.com (P.G.); 17320138484@163.com (M.Z.); wangsc1204@163.com (S.W.); cb960821@163.com (B.C.); liumeng1710@163.com (M.L.); zyh1306228674@163.com (Y.Z.); jiajufang365@163.com (J.J.); zhangqq2020123@163.com (Q.Z.); zhangs9426@163.com (B.Z.); 13298176428@163.com (R.W.); 2 The Engineering and Technology Center for Chinese Medicine Development of Henan Province, 156 Jinshui East Road, Zhengzhou 450046, China

**Keywords:** eriodictyol, homoeriodictyol, NLRP3 inflammasome, Alzheimer’s disease, Aβ_25–35_

## Abstract

(1) Alzheimer’s disease (AD) is a neurodegenerative disorder, and it is now widely accepted that neuroinflammation plays a key role in its pathogenesis. Eriodictyol (Eri) and homoeriodictyol (Hom), dihydroflavonoids extracted from a variety of plants, have been confirmed to display a relationship with neuroprotection. (2) Methods: An AD mouse model was constructed by intracerebroventricular (ICV) injection of the Aβ_25–35_ peptide, and Eri and Hom were administered orally for 4 weeks. UPLC-MS/MS was used to determine whether Eri and Hom cross the blood–brain barrier to exert their therapeutic effects. Histological changes in the brain and levels of Aβ were evaluated, and Y-maze and new object recognition experiments were conducted to assess the effects of Eri and Hom on Aβ_25–35_-induced memory impairment in mice. The levels of oxidative stress and apoptosis in peripheral immune cells and progenitor cells in the hippocampal region were analyzed by flow cytometry and in vitro assays. Western blotting and enzyme-linked immunosorbent assays (ELISA) were used to measure the expression levels of NLRP3 inflammasome-related proteins and inflammatory factors in the brain. The effect of nigericin (an agonist of the NLRP3 inflammasome) on Eri and Hom intervention in LPS-induced N9 microglia was examined using a High Content Screening System. (3) Results: Eri and Hom reduced neuronal damage in mouse brain tissue, decreased Aβ levels in the brain, downregulated oxidative stress and apoptosis levels, and improved learning and memory capacity by crossing the blood–brain barrier to exert its effects. Moreover, Eri and Hom inhibited NLRP3 inflammasome activation and ameliorated immune cell disorder. Furthermore, the effect of Eri and Hom on LPS-induced N9 microglia disappeared after the addition of nigericin to agonize NLRP3 receptors. (4) Conclusions: Eri and Hom improved Aβ_25–35_-induced memory impairment in mice by inhibiting the NLRP3 inflammasome.

## 1. Introduction

Alzheimer’s disease (AD) is a neurodegenerative disorder and one of the most common types of dementia. AD patients are characterized by progressive memory loss, cognitive decline, and abnormal mental behavior [1,2]. The most common pathological feature in the brain of AD patients is the accumulation of amyloid Aβ plaques and intracellular neurofibrillary tangles (NFTs) composed of hyperphosphorylated p-Tau protein [3,4]. These two types of oligomers are harmful to neurons and can cause neuroinflammation and oxidative stress in the brain, ultimately leading to synaptic dysfunction and neuronal loss [5,6]. Moreover, stimulated by Aβ plaques, microglia-specific nucleotide-binding oligomerization domain (NOD)-like receptor protein 3 (NLRP3) inflammasome-mediated chronic neuroinflammation is involved in the pathogenesis of AD [7,8]. The NLRP3 inflammasome is the most characteristic inflammasome of AD, which comprises a sensor protein (NLRP3), an adaptor protein (ASC: apoptosis-associated speck-like protein containing a caspase activation and recruitment domain), and an effector protein (caspase-1) [9]. Activation of the NLRP3 inflammasome causes the generation of caspase-1-mediated interleukins, IL-1β and IL-18, in microglia, which further aggravates the development and progression of AD [10]. Therefore, the NLRP3 inflammasome may be an important molecular target for the treatment of AD by regulating neuroinflammation.

The modulation of inflammatory responses is a promising therapeutic strategy in AD. The main drugs currently used to treat AD are non-steroidal anti-inflammatory drugs (NSAIDs), such as indomethacin; however, they lack distinct effects and have unfavorable side-effect profiles in long-term treatment [11]. Therefore, there is a huge demand for novel drugs to treat AD. Natural medicinal plants have great neuroprotective potential, and the use of these natural products in the treatment of AD by modulating the NLRP3 inflammasome is very promising.

Eriodictyol (Eri) and homoeriodictyol (Hom) are two dihydroflavonoids that occur widely in plants. According to previous reports, Eri has a variety of therapeutic effects including neuroprotective [12,13,14], antioxidant [15], and anti-inflammatory actions [16]. and Hom has antioxidant [17], anti-inflammatory [18], and anti-tumor effects [19]. In addition, it has been shown that flavonoids have an inhibitory effect on the formation of the NLRP3 inflammasome [20]. However, it remains unclear whether Eri and Hom will successfully treat AD by inhibiting the NLRP3 inflammasome. The aim of the present research is to investigate whether Eri and Hom can improve Aβ_25–35_-induced memory impairment in mice by modulating the NLRP3 inflammasome.

## 2. Results

### 2.1. Eri and Hom Cross the Blood–Brain Barrier

**Figure 1:**
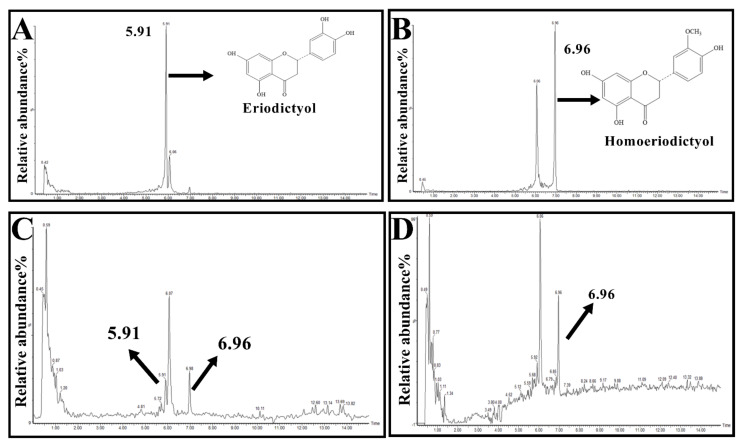
Content of Eri and Hom in the brain detected by UPLC-MS/MS. (**A**). The chromatogram of eriodictyol. (**B**). The chromatogram of homoeriodictyol. (**C**). Brain tissue samples of eriodictyol. (**D**). Brain tissue samples of homoeriodictyol.

The direct action of drugs on the central nervous system (CNS) presupposes that the blood must first cross the blood–brain barrier (BBB) for the drug to be effective [21]. The content of Eri and Hom in the brain was analyzed by UPLC-MS/MS, and the retention time of Eri and Hom was 7.5 ng/g and 205.3 ng/g, respectively, indicating that these compounds were able to cross the BBB. Hom was also detected in the brain of mice given Eri (Figure 1 and Table 1).

### 2.2. Eri and Hom Improve Aβ_25–35_-Induced Memory Impairment and Cognitive Dysfunction in Mice

**Figure 2:**
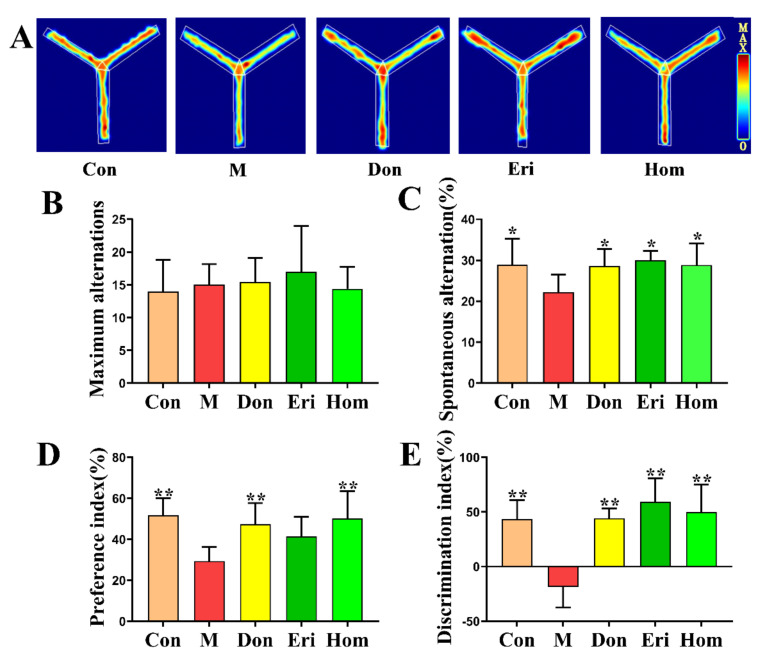
Eri and Hom improve Aβ_25–35_-induced memory impairment and cognitive dysfunction in mice. (**A**–**C**) Observed Y-maze autonomous alternation behavior of mice and maximum number of arm advances and autonomous alternation rate. (**D**,**E**) In the new object discrimination experiment, we calculated the priority coefficient and discrimination coefficient of mice at 24 h. The data were expressed as the mean ± SD. *n* = 8, * *p* < 0.05, ** *p* < 0.01 compared with the M group. Con means the control, M means the model, Don means the donepezil, Eri means the eriodictyol, Hom means the homoeriodictyol.

To confirm the effects of Eri and Hom on AD model mice, we used the Y-maze test and the NOR experiment to assess memory and cognitive function in mice [22,23]. Within 5 min of the Y-maze experiment, the autonomous alternation rate of the model group was significantly lower than that of the control group, and the autonomous alteration rate of mice in the treatment groups was significantly higher than that of the model group (Figure 2C, *p* < 0.01); however, there was no significant difference at maximum arm entries between the groups (Figure 2B). In the NOR experiment, we found that the index of preference and dislike for new objects of mice in the model group was significantly reduced; however, treatment with Eri and Hom, similar to the positive control Don, significantly increased the preference for new objects (Figure 2D,E, *p* < 0.01).

### 2.3. Eri and Hom Alleviate Hippocampal Neuronal Damage Induced by Aβ_25–35_

**Figure 3:**
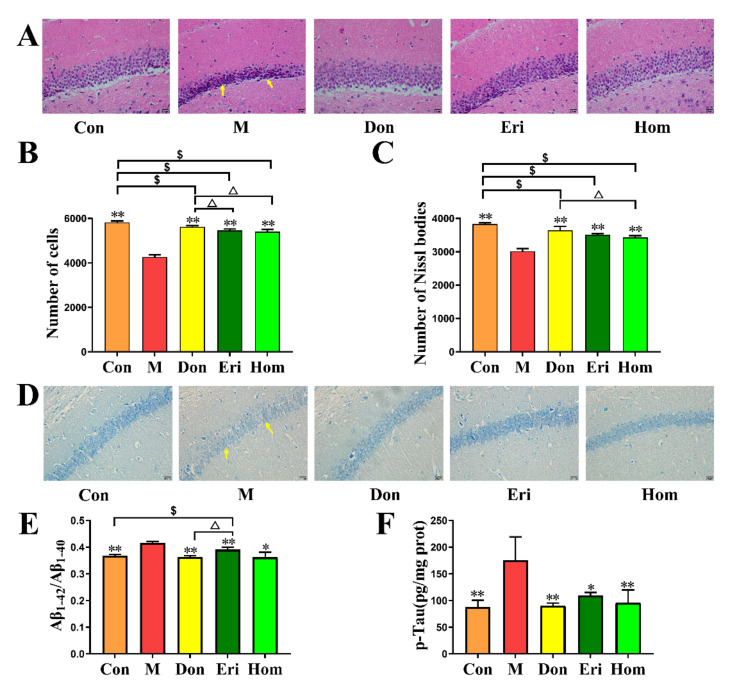
Effects of Eri and Hom on the hippocampus tissue of mice induced by Aβ_25–35_. (**A**) Pathological section of the hippocampus of mice in various groups (20 μm). (**B**) The number of hippocampal cells in each group of mice. (**C**) The number of the hippocampus in each group, *n* = 5. (**D**) Nissl staining in the hippocampus of mice in various groups (20 μm). (**E**,**F**) The levels of Aβ_1–42_/Aβ_1–40_ and p-Tau in mice hippocampus, *n* = 4. The data were expressed as the mean ± SD., * *p* <0.05, ** *p* < 0.01 compared with the M group, △ *p* < 0.05 compared with the Don group, $ *p* < 0.05 compared with the Con group. The yellow arrows in the figure indicate damaged neuronal cells. Con means the control, M means the model, Don means the donepezil, Eri means the eriodictyol, Hom means the homoeriodictyol.

The results of hematoxylin and eosin (H&E) and Nissl staining show that after intracerebral injection of Aβ_25–35_, hippocampal neuronal cells were disordered in arrangement, some cells were deeply stained, solidly shrunken, and rod-shaped, or triangular (Figure 3A), hippocampal neurons appeared atrophied, and a large number of Nissl bodies were lost (Figure 3D). After intervention administration, the number of neuronal cells was significantly restored (Figure 3B, *p* < 0.01), the arrangement was tighter and more regular, and the number of Nissl bodies was increased (Figure 3C, *p* < 0.01). Compared with the Con group, the three administration groups had different degrees of pathological damage, and Don is better than Eri and Hom in improving pathological damage. Aβ is a key pathological product that induces AD [24], and p-Tau protein is also considered to be a biomarker of AD [25]. Don, Eri, and Hom significantly decreased the levels of Aβ_1–40_, Aβ_1–42_, and p-Tau in the hippocampus (Figure 3E,F, *p* < 0.01).

### 2.4. Eri and Hom Reduce Oxidative Stress in the Brain of Aβ_25–35_-Induced Mice

**Figure 4:**
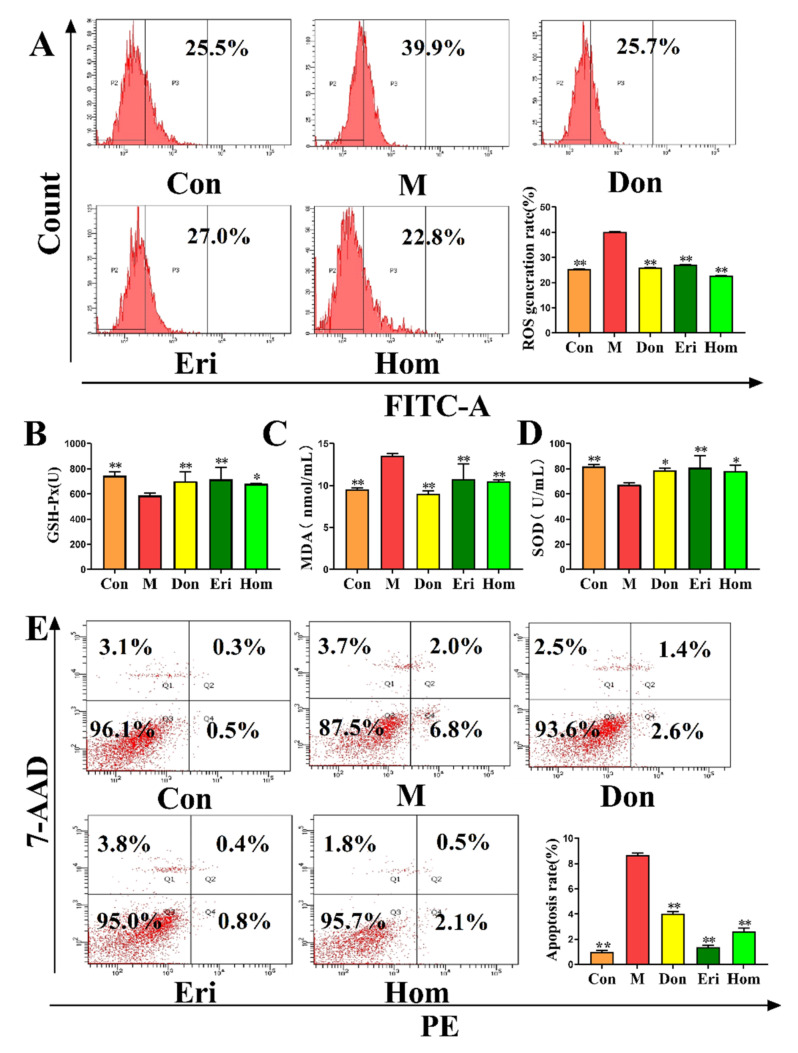
Eri and Hom reduce the oxidative stress in the brain tissue of mice injected with Aβ_25–35_. (**A**) The levels of ROS in primary mouse brain cells, and quantitative analysis of ROS. *n* = 3. (**B**–**D**) The levels of GSH-Px, MDA, and SOD in serum, *n* = 4. (**E**) The levels of apoptosis in primary mouse brain cells, and quantitative analysis of apoptosis, *n* = 3. The data were expressed as the mean ± SD. * *p* < 0.05, ** *p* < 0.01 compared with the M group. Con means the control, M means the model, Don means the donepezil, Eri means the eriodictyol, Hom means the homoeriodictyol.

Studies have shown that oxidative stress is closely related to AD [26]. We measured the levels of ROS (Figure 4A, *p* < 0.01) and apoptosis (Figure 4E, *p* < 0.01) in primary hippocampal cells and found that Eri and Hom reduced the levels of both. In addition, the levels of GSH-Px (Figure 4B, *p* < 0.05), MDA (Figure 4C, *p* < 0.01), and SOD (Figure 4D, *p* < 0.05) in the serum were measured, and we found that the levels of GSH-Px and SOD in the Eri and Hom groups were significantly increased, while the level of MDA was significantly decreased. In addition, Don, as a positive control, was found to have beneficial effects.

### 2.5. Eri and Hom Regulate the Level of Immune Cells in Aβ_25–35_-Induced Mice

**Figure 5:**
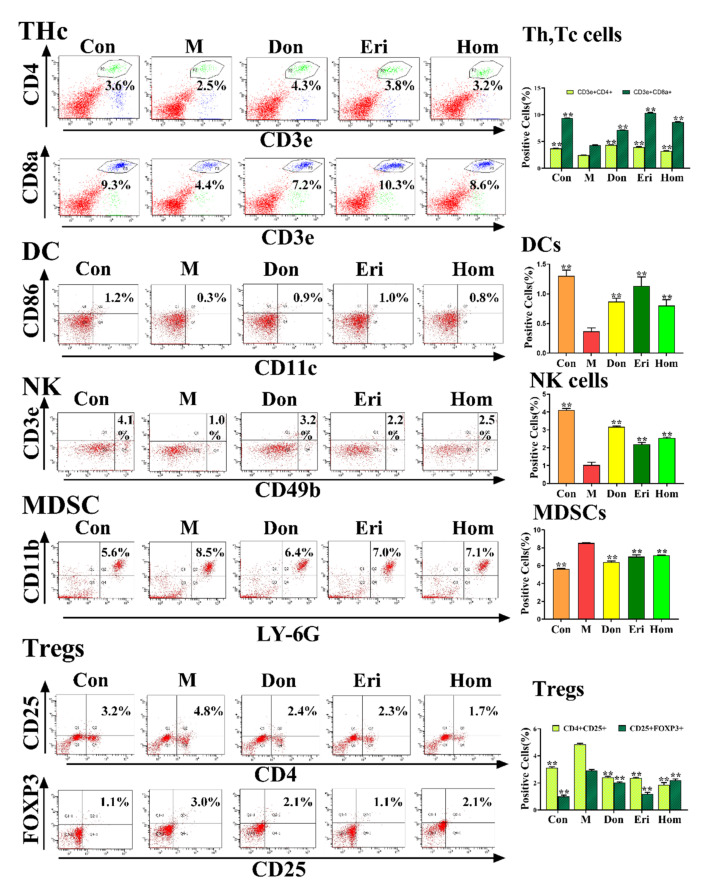
Eri and Hom regulate the level of immune cells in mice injected with Aβ_25-35._ Flow cytometry detected Th cells (CD3e+, CD4+), Tc cells (CD3e+, CD8a+), NK cells (CD49b+, CD3e+), DCs cells (CD11c+, CD86+), Tregs cells (CD4+, CD25+, FOXP3+), and MDSC cells (LY6G+, CD11b+) in the peripheral blood of mice. The immune cell quantification results are shown on the right side of the figure above; the data were expressed as the mean ± SD. *n* = 3, ** *p* < 0.01 compared with the M group. Con means the control, M means the model, Don means the donepezil, Eri means the eriodictyol, Hom means the homoeriodictyol.

Studies have demonstrated that immune cells are a key factor in the neuroinflammatory response that promotes the degenerative process of AD [27]. We analyzed the immune cells in peripheral blood and splenic tissues and found a significant increase in the number of NK cells, Th cells, Tc cells, and DCs, and a significant decrease in MDSCs and Treg cells in mice injected intracerebroventricularly with Aβ_25–35_. Administration of Eri and Hom resulted in increased numbers of NK cells, Th cells, Tc cells, and DCs, and decreased numbers of MDSCs and Treg cells in peripheral blood (Figure 5, *p* < 0.01) and splenic tissues (Figure S1, *p* < 0.01). Moreover, Don also had a favorable effect on the regulation of immune cells.

### 2.6. Eri and Hom Inhibit NLRP3 Inflammasome Activation

**Figure 6:**
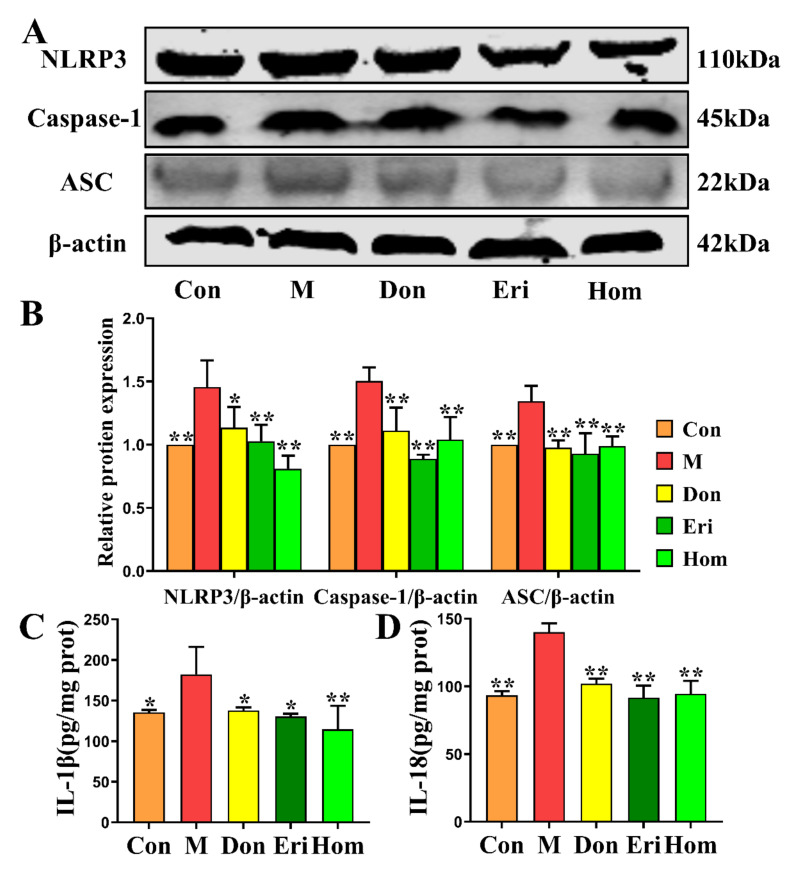
Eri and Hom inhibit NLRP3 inflammasome activation. (**A**). Expression of NLRP3, Caspase-1, and ASC proteins in brain tissue (**B**) Statistical analysis of relative expression of NLRP3, Caspase-1, and ASC. (**C**,**D**) Measure the levels of IL-1β and IL-18 in the brain tissue according to ELISA kits. The data were expressed as the mean ± SD. *n* = 3 * *p* < 0.05, ** *p* < 0.01 compared with the M group. Con means the control, M means the model, Don means the donepezil, Eri means the eriodictyol, Hom means the homoeriodictyol.

The NLRP3 inflammasome has been confirmed to be closely related to AD [10,28]. Results of near-infrared in vivo imaging show that Eri and Hom have excellent anti-inflammatory effects (Figure S2, *p* < 0.05). By measuring the levels of NLRP3 inflammasome-related proteins in brain tissue, we found that Eri and Hom reduced the protein expression levels of NLRP3, Caspase-1, and ASC, and the inflammatory factors IL-18 and IL-1β in brain tissue (Figure 6A–D, *p* < 0.05). In addition, Don also had a positive effect (Figure 6A–D, *p* < 0.05).

### 2.7. Eri and Hom Inhibit N9 Microglia via the NLRP3 Inflammasome

**Figure 7:**
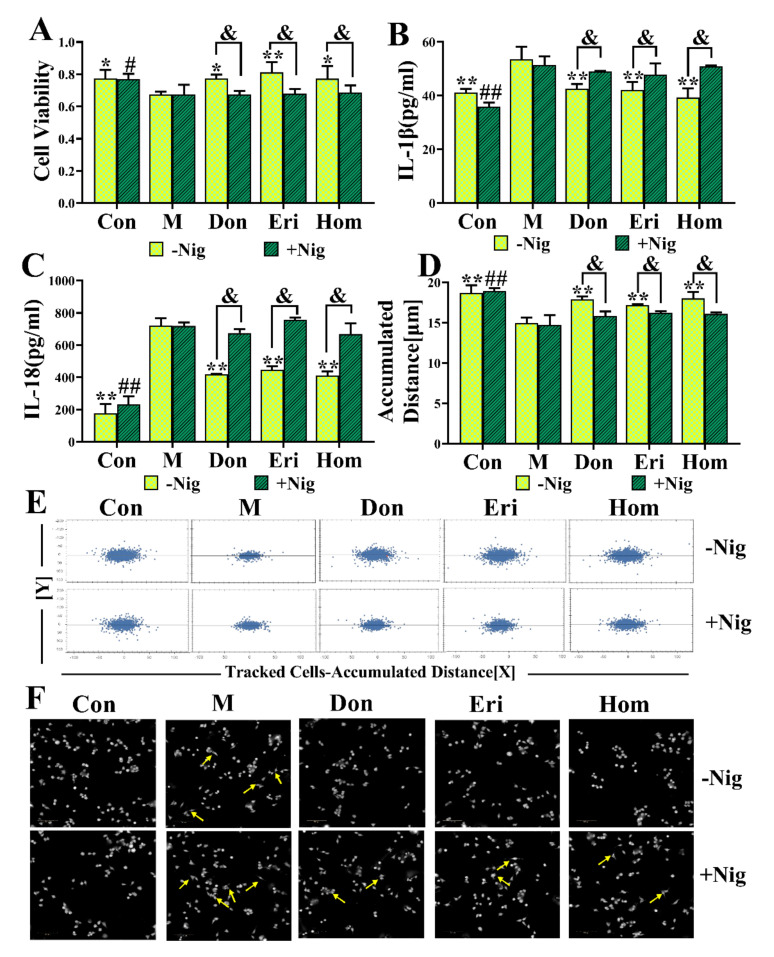
Eri and Hom inhibit N9 microglia via the NLRP3 inflammasome. (**A**) Cell viability was measured by MTT assay. N9 cells were treated with the Donepezil (10 μmol/L + 1 ug/mL LPS) group; Eri (10 μmol/L + 1 ug/mL LPS) group; and Hom (10 μmol/L + 1 ug/mL LPS) group; this effect disappeared after Nigericin (10 μmol/L) stimulation, *n* = 4. (**B**,**C**) Measure the levels of IL-1β and IL-18 in the supernatant of the N9 cells according to the instructions of the kit, *n* = 4. (**D**–**F**) N9 cells migration ability and morphology changes detected via High Content Screening System, *n* = 6. The data were expressed as the mean ± SD. * *p* < 0.05, ** *p* < 0.01 compared with the M group; # *p* <0.05, ## *p* < 0.01 compared with the M group (Add Nigericin). & *p* < 0.05, Compared with the agonist group. Con means the control, M means the model, Don means the donepezil, Eri means the eriodictyol, Hom means the homoeriodictyol, Nig means the nigericin.

To further determine the regulatory effect of Eri and Hom on the NLRP3 inflammasome, N9 microglial cells were selected for culture in vitro. We set out to explore whether the role of Eri and Hom still exists by adding an agonist of the NLRP3 inflammasome. The NLRP3 agonist, nigericin (20 μL,10 μmol/L), was administered 30 min before treatment with Eri and Hom to evaluate whether the observed effects elicited by Eri and Hom were mediated via the NLRP3 inflammasome. The results show that Eri and Hom restored N9 cell viability (Figure 7A, *p* < 0.05) and migration levels (Figure 7D–F, *p* < 0.05) and reduced the levels of inflammatory factor (Figure 7B,C, *p* < 0.05); these effects disappeared when the nigericin was added. Moreover, Don had the same effect.

## 3. Discussion

AD has been recognized by the World Health Organization as a global public health priority; however, to date, there remains no effective treatment. The aggregation of toxic Aβ fragments is considered the main cause of cognitive dysfunction [29]. In addition, hyperphosphorylation of the microtubule-associated protein, tau, in neurons occurs downstream of the excessive accumulation of Aβ, which eventually leads to dementia [30]. Aβ induction is one of the most commonly used methods to establish animal models of AD. Aβ_25–35_ is the shortest fragment capable of forming large β-sheet fibrils and retaining the toxicity of full-length Aβ _(1–40/42)_ peptides [31]. In the present study, mice injected with Aβ_25–35_ were used as an in vivo AD model to explore the biological activity of Eri and Hom. Don was selected as a positive control for the treatment of AD, since it can improve cognitive function in AD patients [32,33]. Eri and Hom were found to be able to cross the blood–brain barrier and be detected in brain tissue by UPLC-MS/MS, laying the foundation for their effectiveness (Figure 1). In our results, we found that Hom was detected in the brains of mice treated with Eri, and we speculated that Eri was metabolized to Hom by methylation in the brain, and this phenomenon will be studied in the future. The Y-maze measures the tendency to explore the environment and short-term spatial memory through the prefrontal cortex, and new object recognition is commonly used to assess the formation of general learning and memory abilities [22,34]. In this study, Eri and Hom were found to improve short-term spatial memory in mice as well as the ability of mice to recognize new objects. The hippocampus and cerebral cortex are important systems related to memory, and neuronal damage in these areas leads to cognitive impairment [35]. Therefore, we further examined the neuronal structure of the mouse cerebral cortex and hippocampus. H&E and Nissl pathological changes revealed neuronal abnormalities and loss of Nissl bodies in the cerebral cortex and hippocampus. Treatment with Eri and Hom ameliorated these lesions and reduced neuronal loss (Figure 3). Aβ deposition and hyperphosphorylated p-Tau protein are key pathological changes in AD, and intervention with Eri and Hom reduced the amount of amyloid and p-Tau in the brains of mice injected with Aβ_25–35_ (Figure 3). These results confirm that Eri and Hom ameliorated Aβ_25–35_-induced pathological changes in the brain and thus improved memory impairment. In addition, the present study shows that the recognition and memory abilities of mice injected with Aβ_25–35_ were significantly improved following the administration of Don.

Under oxidative stress conditions, in vivo oxidative defense protein inhibitors can bind to NLRP3 and promote the formation and activation of inflammatory vesicles [36]. Excessive oxidation also leads to inflammatory infiltration of neutrophils and a large number of reactive oxygen species (ROS), which directly participate in the regulation of cell survival and apoptosis [37]. Moreover, intervention with Eri and Hom in the present study led to reduced levels of oxidative stress and apoptosis in the hippocampus of mice injected with Aβ_25–35_ (Figure 4). In recent years, growing evidence has emerged that the NLRP3 inflammasome plays a central role in the pathogenesis of neuroinflammation-mediated AD [38,39,40]. In the present study, in vivo imaging of mice revealed that Eri and Hom have excellent anti-inflammatory effects (Figure S2). Studies have demonstrated that when immune cells are unstimulated, an adaptor protein (apoptosis-associated speck-like protein, or ASC) is located in the nucleus; however, following activation by pathological stimuli (such as neuronal damage or protein aggregation), ASC translocates to the cytoplasm and induces binding of the thermal domain of the NLRP3 inflammasome to ASC and recruitment of pro-Caspase-1 through self-cleavage to produce activated Caspase-1. Caspase-1 is cleaved, processes the downstream interleukin-1β precursor (pro-IL-1β), and is released extracellularly [41,42]. Subsequently, IL-1β and IL-18 activate numerous signaling pathways and induce inflammatory responses that lead to neuronal damage or death [43]. The expression levels of proteins and inflammatory factors associated with the NLRP3 inflammasome were reduced following intervention with Eri and Hom (Figure 6). We speculate that Eri and Hom may ameliorate the inflammatory damage induced by intracerebral injection of Aβ_25–35_ by inhibiting the NLRP3 inflammasome.

Furthermore, there is increasing evidence that immune cells, such as Thc, Tregs, DCs, NKs, and MDSCs are closely related to the development of AD, and that immune mechanisms contribute to the pathogenesis of AD [44,45,46,47,48]. The immune response leads to activation of the inflammasome and further release of inflammatory mediators, exacerbating the progression and severity of AD [49]. It has been suggested that the dysfunction of peripheral immune cell subpopulations leads to deterioration of the immune environment in the central nervous system (CNS) [50]. In our experiments, intracerebral injection of Aβ_25–35_ in mice produced significant changes in Thc, Tregs, DCs, NKs, and MDSCs in the peripheral blood (Figure 5) and spleen (Figure S1), as well as a significant increase in the levels of the NLRP3-related inflammatory factors, IL-18 and IL-1β, in the brain (Figure 6). Moreover, a close relationship between DCs and IL-18 and IL-1β has been found in the literature [51,52,53]. In summary, Eri and Hom improve immune cell levels in the peripheral blood and spleen, thus reducing inflammation in the brain.

To further investigate the anti-AD effects of Eri and Hom through inhibition of the NLRP3 inflammasome, N9 microglia were subjected to in vitro experiments with the addition of nigericin, which is an agonist of the NLRP3 inflammasome [54,55]. The results show that Eri and Hom promoted microglial proliferation and migration by inhibiting the NLRP3 inflammasome and reduced IL-1β and IL-18 production, and that these effects disappeared following the addition of nigericin (Figure 7). These data reinforce the suggestion that Eri and Hom improve Aβ_25–35_-induced memory impairment by inhibiting the NLRP3 inflammasome to reduce inflammation.

## 4. Materials and Methods

### 4.1. Experimental Animals

Male Kunming mice (KM; 7 weeks old) were obtained from Beijing Vitalstar Biotechnology Co., Ltd (Beijing, China). (*n* = 50) and housed at a constant temperature (25 ± 2 °C) and relative humidity (60 ± 10%) with a 12 h light/dark cycle and free access to food and water. All procedures were performed according to the Guidelines for Care and Use of Laboratory Animals of the Henan University of Chinese Medicine, and experiments were approved by the Animal Ethics Committee of the Henan University of Chinese Medicine.

### 4.2. Treatment Schedule

A total of 50 mice were randomly divided into five groups: control (Con), model (M), donepezil-treated (positive control, 10 mg/kg/d, Don), eriodictyol-treated (10 mg/kg/d, Eri), and homoeriodictyol-treated (10 mg/kg/d, Hom). Aβ_25–35_ was injected into the hippocampus of mice in all groups except the control group, the control mice were injected with an equal amount of saline in the brain. The Aβ_25–35_ peptides (Sangon Biotech, Shanghai, China) were aggregated by incubation in distilled water (1 mg/mL) at 37 °C for 7 days and subsequently diluted to the final concentration in sterile saline immediately before the experiment. The mice were anesthetized with isoflurane gas and placed in a stereotactic apparatus (RAW Life Science, Shenzhen, China). A microinjection needle was positioned 2 mm posterior to the bregma, 2.2 mm lateral, and 1.5 mm inferior to the bone surface for ICV injection [31,56,57]. Aβ_25–35_ in saline (300 μmol/L) was injected for 15 min, with the needle kept in place for 5 min before and after injection. Donepezil hydrochloride tablets (21030009, Zein Biotechnology, Chongqing, China), eriodictyol (CAS:552-58-9, PhytoUnico, Chengdu, China), and homoeriodictyol (CAS:446-71-9, PhytoUnico, Chengdu, China) were administered by gavage, beginning two weeks after surgical intervention, and continued for the subsequent 4 weeks. Equal amounts of saline were given by gavage in all groups except for the drug administration group. A series of behavioral tests was started three weeks after drug administration.

### 4.3. Behavioral Analysis

The Y-maze test is mostly used to evaluate spatial learning and memory abilities in mice. This experiment was conducted using the YMT-100 experimental analysis system (Taimeng Software Co., Ltd., Chengdu, China). During the experiment, the mice were placed in the central triangular area and allowed to explore freely for 5 min. The sequence of mice entering each arm and the total number of arms entered were recorded. If the animal entered three different arms consecutively, it was considered an alternation, and the maximum arm entrance is defined as N–2; alternation percentage (%) = alternation / (N–2) × 100% [22,58].

The new object recognition (NOR) experiment is a test method to evaluate learning and memory abilities in mice based on the innate characteristics of rodents. In this experiment, two identical objects, A1 and A2, were placed on the left and right ends of the square area, respectively. The mouse was then placed at a position equidistant from each object, and the number of times exploring the new object within 5 min (both feet touched the new object once) was recorded. After 24 h, A2 was replaced with another new object B, and the number of times exploring the new object within 5 min was recorded [34,59].

### 4.4. Pharmacokinetic Study of Eriodictyol and Homoeriodictyol in Mouse Brain Tissue

Brain tissue homogenate was prepared as follows: 0.1 g tissue was weighed, a 4-fold volume of saline was added, and the sample was centrifuged at 3000 rpm for 10 min. A 200 μL aliquot of the upper layer of the homogenate was removed, 20 μL quercetin (internal standard) solution (100 ng/mL) was added, and the solution was vortexed for 30 s. A 600 µL aliquot of acetonitrile was added to precipitate the protein, and the solution was vortexed for 5 min and centrifuged at 12,000 rpm for 10 min. The supernatant was carefully aspirated, transferred to a heart centrifuge tube, and dried at 45 °C under nitrogen. The sample was analyzed by UPLC-MS/MS, and the peak area was quantitated using the internal standard method.

Chromatographic separation was achieved on an ACQUITY UPLC BEH C_18_ column (2.1 mm × 50 mm, 1.7 μm) at 35 °C. The mobile phases consisted of acetonitrile (A) and 0.1% formic acid in water (B) using a gradient elution according to the following profile: 0–1.2 min, 2–10% A; 1.2–2.52 min, 10–15% A; 2.52–4.32 min, 15–20% A; 4.32–6.12 min, 20–30% A; 6.12–7.32 min, 30–35% A; 7.32–9.12 min, 35–40% A; 9.12–10.92 min, 40–60% A; 12 min, 2% A. The equilibration time of the gradient elution was 5 min; the run time was 15 min; the injection volume was 5 µL, and the flow rate was set at 0.3 mL/min. Mass spectrometry was conducted on a Waters Xevo TQD triple-quadrupole mass spectrometer with an electrospray ionization source. The negative ionization mode was used, and the ions were monitored in the multiple reaction monitoring (MRM) mode. The ESI source parameters were as follows: capillary voltage: 2800 V; source temperature: 150 °C; solvent gas temperature: 350 °C; solvent airflow: 650 L/h; nebulizer pressure: 3.0 V. The mass spectral parameters and retention times of the three compounds in MRM mode are shown in Table 2.

### 4.5. Histopathology of Brain Tissues

Brain tissue was fixed in 4% paraformaldehyde, dehydrated in increasing concentrations of alcohol, and embedded in paraffin. Specimens were cut into 4 μm sections using a section cutter and stained with hematoxylin–eosin (H&E, Servicebio Technology Co., Ltd., Wuhan, China) and Nissl stain (Servicebio Technology Co., Ltd., Wuhan, China) according to the manufacturer’s instructions. The number of cells in the hippocampus was quantitated using the StrataQuest software (version 7.0.1, TissueGnostics GmbH, Vienna, Austria).

### 4.6. Biochemical Indexes Assay

Plasma was collected, and the levels of total superoxide dismutase (SOD, A001-3-2, Nanjing Jiancheng Bioengineering Institute, Nanjing, China), malondialdehyde (MDA, A003-1-2, Nanjing Jiancheng Bioengineering Institute, Nanjing, China), and glutathione peroxidase (GSH-Px, A005-1-2, Nanjing Jiancheng Bioengineering Institute, Nanjing, China) were measured using the corresponding kit according to the manufacturer’s instructions. The levels of Aβ_1–40_ (E-EL-M300, Elabscience Biotechnology Co., Ltd., Wuhan, China), Aβ_1–42_ (E-EL-M3010, Elabscience Biotechnology Co., Ltd., Wuhan, China), p-Tau (E-EL-M1289c, Elabscience Biotechnology Co., Ltd., Wuhan, China), IL-1β (E-EL-M0037c, Elabscience Biotechnology Co., Ltd., Wuhan, China), and IL-18 (E-EL-M0730c, Elabscience Biotechnology Co., Ltd., Wuhan, China) in the supernatant from the brain homogenate were assessed using the corresponding enzyme-linked immunosorbent assay (ELISA) kits following the manufacturer’s protocols.

### 4.7. Flow Cytometry Analysis of Immune Cells

The spleen was quickly dissected, an appropriate amount of tissue and PBS was placed into a 70 μm filter, and the tissue was gently ground clockwise using a grinder until the tissue disappeared. The filtrate was collected and centrifuged at 1500 rpm for 5 min, after which the supernatant was carefully discarded to obtain primary splenic cells. The original generation of splenic cells was resuspended in 500 μL PBS, and the suspension was evenly distributed among 5 flow cytometry tubes labeled natural killer cells (NKs), dendritic cells (DCs), helper and cytotoxic T cells (Thc), regulatory T cells (Tregs), and myeloid-derived suppressor cells (MDSCs). The corresponding antibody was added to each tube and incubated for 30 min in the dark, after which 1× red blood cell lysate was added and allowed to lyse for approximately 10 min until the sample became clear. The sample was then centrifuged at 300× *g* for 5 min, and the supernatant was discarded. The cells were resuspended in 2 mL PBS and centrifuged (repeated twice), and 300 μL PBS was added to each tube. The cells were then analyzed by flow cytometry (556547, BD FACSAria III, New York, NY, USA). Tregs needed to be ruptured before staining with the anti-Foxp3 antibody. The results of this section are displayed in the Supplementary Materials Figure S1.

Blood was collected from mouse eyeballs, and the plasma was evenly distributed (100 μL each) among 5 flow cytometry tubes labeled NKs, DCs, Thc, Tregs, and MDSCs. The staining method used was the same as that for the primary splenic cells. The cells were then analyzed by flow cytometry (556547, BD FACSAria III, New York, NY, USA).

### 4.8. Flow Cytometry Analysis of Reactive Oxygen Species (ROS) and Apoptosis Markers in Primary Brain Cells

Following dissection, the hippocampus was placed in a centrifuge tube containing 1 mL cold PBS, cut into pieces using scissors, and filtered through a 70 μm mesh by washing several times. The filtrate was collected and centrifuged at 1200 rpm for 5 min, and the supernatant was discarded. The cells were resuspended in a solution containing the fluorescent probe 2′,7′-dichlorofluorescein diacetate (DCFH-DA, 10 μmol/L, CA1410, Solarbio Science & Technology Co., Ltd., Beijing, China), incubated at 37 °C for 20 min, and washed 3 times with PBS. The fluorescence intensity was measured by flow cytometry (BD FACSAria III, New York, NY, USA) to detect reactive oxygen species (ROS) [60].

A PE Annexin V apoptosis detection kit (BD Biosciences, New York, NY, USA) was used to evaluate the level of apoptosis. The collected primary brain cells were resuspended in 100 μL loading buffer, and 5 μL each PE Annexin V and 7-Amino-Actinomycin (7-AAD) were added. After vortexing, cells were incubated in the dark at room temperature for 15 min and subsequently analyzed by flow cytometry (556547, BD FACSAria III, New York, NY, USA).

### 4.9. Western Blot Analysis

The hippocampus was carefully dissected and placed in lysis buffer for homogenization. After centrifugation, the supernatant was aspirated and a BCA protein assay kit (Solarbio, Life Science, Beijing, China) was used to determine the protein concentration. Proteins were separated by SDS-PAGE, transferred to polyvinylidene fluoride membrane, blocked with 5% bovine serum albumin (BSA, 4240GR100, BioFroxx, Guangzhou, China), and incubated at 4 °C overnight with the following primary antibodies: NLRP3 (1:1000, ab4207, Abcam), ASC (1:1000, #67824, Cell Signaling Technology), Caspase-1 (1:1000, ab1872, Abcam), and β-actin (1:5000, AC026, Abclonal, Wuhan, China). After washing in PBS, the membranes were incubated with Alexa Fluor^®^-labeled fluorescent secondary antibodies for 90 min and then analyzed using an Odyssey^®^CLx near-infrared imaging system (Li-COR Biosciences, Lincoln, NE, USA).

### 4.10. Near-Infrared In Vivo Imaging

A substitute for glucose, 2-DG, is taken up by inflammatory tissue and can be quantitated using a near-infrared fluorescence imaging system (Pearl, Li-COR Biosciences, Lincoln, NE, USA). One hour after the intraperitoneal injection of LPS in mice, a 10 nmol IRDye 800CW 2-DG optical probe (C71103-07, LI-COR Biosciences, Lincoln, NE, USA) was injected into the tail vein. Twenty-four hours later, a small animal live imaging system was utilized to monitor inflammation in the mice [61]. The results of this section are displayed in the Supplementary Materials Figure S2.

### 4.11. Cell Culture and Treatment

Mouse N9 microglial cells were purchased from Otwo Biotech (Shenzhen) Inc. (Shenzhen, China) and cultured in Dulbecco’s modified Eagle’s medium (DMEM) supplemented with 2 mmol/mL L-glutamine, 50 units/mL penicillin, 50 μg/mL streptomycin, and 10% heat-inactivated fetal bovine serum (FBS) at 37 °C and 5% CO_2_.

### 4.12. Cell Viability Assay

N9 cells were seeded onto 96-well plates (3599, Corning, NY, USA) at 20,000 cells/well and divided into the following groups: control; model (1 μg/mL LPS); donepezil (Don) (10 μmol/L + 1 μg/mL LPS); eriodictyol (Eri) (10 μmol/L + 1 μg/mL LPS); and homoeriodictyol (Hom) (10 μmol/L + 1 μg/mL LPS). In another experiment, the NLRP3 agonist, nigericin (10 μmol/L, 28643-80-3, MedChem Express, Shanghai, China), was added 30 min prior to treatment with LPS, Don, Eri, and Hom. Twenty-four hours later, 20 µL MTT solution (5 mg/mL) was added to each well, and the plates were incubated for 4 h at 37 °C. After discarding the medium, 150 μL DMSO was added to dissolve the purple formazan crystals and the plate was shaken for 10 min. The OD value was then measured at a wavelength of 490 nm on an EPOCH microplate reader (BioTek, Winooski, VT, USA).

### 4.13. Analysis of Cytokines in the Cell Supernatant by ELISA

N9 cells were seeded onto 24-well plates (3524, Corning, NY, USA) at 40,000 cells/well. The cell grouping was consistent with that of the cell viability assay. Twenty-four hours later, the supernatant from the cultured cells was collected and the levels of IL-1β and IL-18 were measured according to the kit instructions.

### 4.14. Cell Migration Assay

The cell migration assay was performed in 96-well plates (E190236X, PerkinElmer, United States) using 20,000 cells/well. The cell grouping was consistent with that of the cell viability assay. The migration dynamics of the cells were monitored for 48 h at 37 °C and 5% CO_2_ by digital phase contrast using the High Content Screening System (Opera Phenix, PerkinElmer, Shanghai, China) [62].

### 4.15. Statistical Analysis

All data were analyzed using the SPSS software version 26.0 and are presented as the mean ± standard deviation. The significant differences between the negative control and the test fractions were assessed by analysis of variance (ANOVA) followed by LSD′s test, or a Student′s *t*-test where appropriate for multiple comparisons. Probability values of 0.05 or less were considered to be statistically significant. Orthogonal partial least squares discriminant analysis (OPLS-DA) was used to calculate the intracerebral content of eriodictyol and homoeriodictyol.

## 5. Conclusions

The current clinical treatments for AD are limited and cannot prevent the progression of the disease. Previous studies have demonstrated that the NLRP3 inflammasome is closely related to AD, and it is thought that its activation in microglia plays a major role in the development of AD. Using both in vitro and in vivo studies, we found that Eri and Hom likely inhibit inflammation through the NLRP3 inflammasome to improve Aβ_25–35_-induced memory impairment in mice. These findings demonstrate the pharmacological effects of Eri and Hom against AD at the molecular level and provide an experimental basis for the development of natural compounds to target AD.

## Figures and Tables

**Table 1:** Linear regression equations and detection limits for eriodictyol and homoeriodictyol.

Compound	Calibration Curve	Correlation Coefficient (R2)	Content (ng/g)
Eriodictyol	Y = 0.109 × X + 0.195	0.992	7.5
Homoeriodictyol	Y = 0.026 × X + 0.057	0.993	205.3

**Table 2:** Optimized mass spectrometry conditions parameters for three compounds, retention time (RT), declusterin pressure (DP), collision energy (CE).

Compound	Ionic Type	RT/min	Parent Ion *m*/*z*	Product Ion *m*/*z*	DP/v	CE/v
Eriodictyol	M-H	5.91	286.9	150.9	36	18
Homoeriodictyol	M-H	6.96	300.9	150.9	42	28
Quercetin	M-H	6.06	300.9	150.9	42	28

## Data Availability

The date presented in this study are available on request from the corresponding author.

## Supplementary Materials

The following supporting information can be downloaded at: https://www.mdpi.com/article/10.3390/molecules27082488/s1, Figure S1: Eri and Hom regulate immune cell levels in the spleen of mice injected with Aβ_25–35_. Figure S2: Eri and Hom inhibit LPS-induced systemic inflammatory responses in animals.
