# Supplementary material for: Eriodictyol and Homoeriodictyol Improve Memory Impairment in Aβ25–35-Induced Mice by Inhibiting the NLRP3 Inflammasome
Source: Molecules. 2022 Apr 12;27(8):2488. doi: 10.3390/molecules27082488 (PMC9025671; doi:10.3390/molecules27082488)
Supplement: Supplementary file 1 [file molecules-27-02488-s001.zip › molecules-1615627-supplementary.pdf]

Supplementary Materials

Eriodictyol and Homoeriodictyol Improve Memory Impairment in Aβ_{25-35}-Induced Mice by Inhibiting the NLRP3 Inflammasome

Pengli Guo [1,2], Mengnan Zeng [1,2], Shengchao Wang [1,2], Bing Cao [1,2], Meng Liu [1,2], Yuhan Zhang [1,2], Jufang Jia [1,2], Qinqin Zhang [1,2], Beibei Zhang [1,2], Ru Wang [1,2], Xiaoke Zheng [1,2,*] and Weisheng Feng [1,2]

1 College of Pharmacy, Henan University of Chinese Medicine, 156 Jinshui East Road, Zhengzhou 450046, China
2 The Engineering and Technology Center for Chinese Medicine Development of Henan Province,156 Jinshui East Road, Zhengzhou 450046, China
* Correspondence: E-mail addresses: zhengxk.2006@163.com (Xiaoke Zheng), fwsh@hactcm.edu.cn (Weisheng Feng)

Eri and Hom regulate immune cell levels in the spleen of mice injected with Aβ_{25-35}.

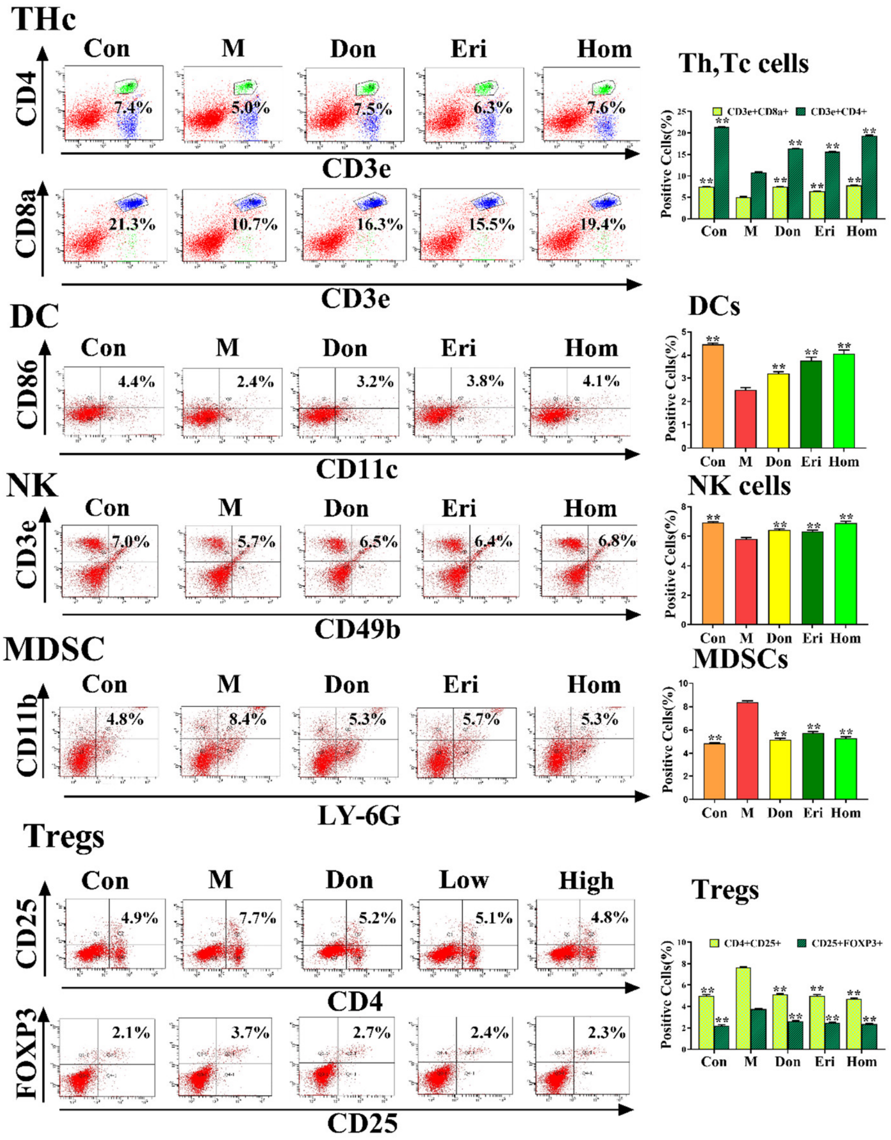

Figure S1. Eri and Hom regulate immune cell levels in the spleen of mice injected with $A\beta_{25\text{-}35}$. Flow cytometry detected Th cells (CD3e+, CD4+), Tc cells (CD3e+， CD8a+), NK cells (CD49b+, CD3e+), DCs cells (CD11c+, CD86+), Tregs cells (CD4+, CD25+, FOXP3+), MDSC cells (LY6G+, CD11b+) in the spleen tissues of mice. The immune cell quantification results are shown on the right side of the figure above. The data were expressed as the mean ± SD. n=3 *p<0.05, **p<0.01 compared with the M group. Con means the control, M means the model, Don means the donepezil, Eri means the eriodictyol, Hom means the homoeriodictyol.

Eri and Hom inhibit LPS-induced systemic inflammatory responses in animals

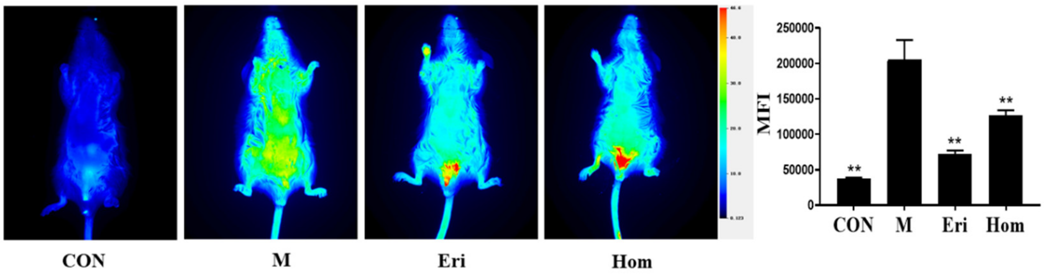

Figure S2. Representative images showed the fluorescent signal of 2-DG probe in mice at 24 h and quantitative result. The data were expressed as the mean ± SD. n=3 *p<0.05, **p<0.01 compared with

the M group. Con means the control, M means the model, Eri means the eriodictyol, Hom means the homoeriodictyol.
